# Editorial: Drug allergy in children

**DOI:** 10.3389/falgy.2024.1530907

**Published:** 2024-12-16

**Authors:** Mara Morelo Rocha Felix, José Laerte Boechat, Fábio Chigres Kuschnir, Mariana C. Castells

**Affiliations:** ^1^ Department of General Medicine, School of Medicine and Surgery, Rio de Janeiro State Federal University, Rio de Janeiro, Brazil; ^2^ Clinical Immunology Service, Internal Medicine Department, Faculty of Medicine, Fluminense Federal University, Rio de Janeiro, Brazil; ^3^ Basic and Clinical Immunology Unit, Department of Pathology, Faculty of Medicine, University of Porto, Porto, Portugal; ^4^ Department of Pediatrics, Faculty of Medicine, Rio de Janeiro State University, Rio de Janeiro, Brazil; ^5^ Division of Rheumatology, Immunology and Allergy, Brigham and Women’s Hospital, Boston, MA, United States

**Keywords:** drug allergy, children, delabeling, beta-lactam antibacterials, chemotherapy - oncology

**Editorial on the Research Topic**
Drug allergy in children

Drug allergy in children is underreported and underdiagnosed, yet most allergy labels occur in infancy and are carried over life unverified. These aspects deserve special considerations and are the objective of this Research Topic. Here we address important features specific to the pediatric age group, such as reaction types, predisposing factors, diagnostic methods, and therapeutic approaches to help healthcare providers with the diagnosis and management of drug allergy in children.

Adverse drug reactions (ADRs) can present unique characteristics due to children's physiology, which differs from that of adults, and can influence drug pharmacokinetics and pharmacodynamics (1). Understanding these differences is key to preventing, identifying, and managing ADRs in young patients.

Diagnosing ADRs in children can pose challenges due to the broad range of clinical manifestations, which can mimic other pediatric conditions (2). Young children may not be able to effectively communicate their symptoms, necessitating a thorough clinical assessment and a high level of suspicion (2).

In the general population 10%–20% of parents report drug allergies in their children, yet, when evaluated, over 90% of cases are not confirmed, leading to unnecessary restrictions (3). Labeling a child as allergic limits options and can lead to less effective, more costly treatments. Most adults claiming to have a drug allergy are labeled in childhood and carry an unverified label thorough their lives, being deprived of important medications at times of need.

Felix et al. address the significant impact of drug hypersensitivity reactions (DHR) in children (Felix et al.). They highlight the challenges of both under-diagnosis and over-diagnosis, complicating clinical practice and public health (Felix et al.). Their review examines recent findings and identifies gaps in pediatric DHR epidemiology, focusing on antibiotic and antiepileptic hypersensitivities, vaccine allergies, and severe cutaneous adverse reactions (SCAR), providing valuable insights for improved pediatric allergy management (Felix et al.).

Sáenz de Santa María et al. discuss the high incidence of delayed-onset skin rashes in children treated with beta-lactam (BL) antibiotics, frequently misclassified as allergic reactions (Sáenz de Santa María et al.). Incorrectly labeling BL allergies in children can lead to unnecessary second-line antibiotic use, increasing adverse effects, promoting multidrug resistance, and extending hospital stays—posing both public health and economic challenges (Sáenz de Santa María et al.). The authors emphasize the importance of “delabeling” to prevent misdiagnoses that persist into adulthood and examine evolving diagnostic practices (Sáenz de Santa María et al.). Recent studies reveal that direct drug provocation testing (DPT) without prior skin or serum tests is a safe, cost-effective approach for low-risk cases in pediatrics. This review explores the latest tools and ongoing debates in BL allergy management in children, aiming to optimize treatment and reduce mislabeling (Sáenz de Santa María et al.).

Kidon et al. discuss DHR in pediatric hemato-oncology patients, who face unique risks due to immune compromise and complex medication regimens (Kidon et al.). Fever, which can signal severe delayed-type hypersensitivity reactions like DRESS or DIHS, may also occur as an isolated reaction to chemotherapeutic agents. The authors present three pediatric cases with intracranial tumors experiencing recurrent fevers following Vinca alkaloid-based chemotherapy (Kidon et al.). These fevers were successfully managed with montelukast, a cysteinyl leukotriene receptor antagonist, allowing uninterrupted completion of chemotherapy regimens. The study suggests montelukast's potential in managing chemotherapy-induced fever in pediatric patients and calls for further research into the underlying mechanisms of drug-induced fever in this population (Kidon et al.).

In conclusion, this Research Topic presents recent developments in pediatric drug allergy diagnosis and treatment, offering crucial insights to enhance pediatric allergy management and optimize care strategies. It provides emphasis in the delabeling process and presents new tools accessible to most care givers. We invite readers to explore these studies, which offer a deeper understanding and effective solutions for the challenges of adverse drug reactions in children.
